# Treatment with 670 nm Light Up Regulates Cytochrome C Oxidase Expression and Reduces Inflammation in an Age-Related Macular Degeneration Model

**DOI:** 10.1371/journal.pone.0057828

**Published:** 2013-02-28

**Authors:** Rana Begum, Michael B. Powner, Natalie Hudson, Chris Hogg, Glen Jeffery

**Affiliations:** 1 Institute of Ophthalmology, University College London, London, United Kingdom; 2 Moorfields Eye Hospital, London, United Kingdom; University of Florida, United States of America

## Abstract

Inflammation is an umbrella feature of ageing. It is present in the aged retina and many retinal diseases including age-related macular degeneration (AMD). In ageing and in AMD mitochondrial function declines. In normal ageing this can be manipulated by brief exposure to 670 nm light on the retina, which increases mitochondrial membrane potential and reduces inflammation. Here we ask if 670 nm exposure has the same ability in an aged mouse model of AMD, the complement factor H knockout (CFH^−/−^) where inflammation is a key feature. Further, we ask whether this occurs when 670 nm is delivered briefly in environmental lighting rather than directly focussed on the retina. Mice were exposed to 670 nm for 6 minutes twice a day for 14 days in the form of supplemented environmental light. Exposed animals had significant increase in cytochrome c oxidase (COX), which is a mitochondrial enzyme regulating oxidative phosphorylation.There was a significant reduction in complement component C3, an inflammatory marker in the outer retina. Vimetin and glial fibrillary acidic protein (GFAP) expression, which reflect retinal stress in Muller glia, were also significantly down regulated. There were also significant changes in outer retinal macrophage morphology. However, amyloid beta (Aβ) load, which also increases with age in the outer retina and is pro-inflammatory, did not change. Hence, 670 nm is effective in reducing inflammation probably via COX activation in mice with a genotype similar to that in 50% of AMD patients even when brief exposures are delivered via environmental lighting. Further, inflammation can be reduced independent of Aβ. The efficacy revealed here supports current early stage clinical trials of 670 nm in AMD patients.

## Introduction

Progressive ageing is associated with systemic inflammation [1]–[3]. This is marked in tissues with high metabolic demand such as the retina that suffers from progressive oxidative stress [4]–[6]. This is partly driven by excess extra-cellular deposition along an ageing Bruch’s membrane, where inflammation becomes a key feature, even in the absence of pathology [2], [7]. It is also a common feature of retinal disease whether it be age related macular degeneration (AMD), diabetic retinopathy or posterior uveitis [8]–[12]. Ameliorating retinal inflammation is becoming a key problem with an ageing population as this may be associated with age related retinal cell loss and declining visual function [13]–[15]. It is also critical in many retinal diseases [5], [10], [11].

There are multiple routes to dealing with retinal inflammation, however key to any success is the need for cheap effective therapies that are minimally invasive and do not require clinical time. Inflammation is partially driven by shifts in mitochondrial function that result in reduced adenosine triphosphate (ATP) production and increased reactive oxygen species (ROS) output [5], [7], [16]. This can be modulated by brief exposure to 670 nm light, which is absorbed by cytochrome c oxidase (COX) and increases ATP production [17], [18]. This has been shown to reduce a range of retinal inflammatory markers in normal aged mice when directly exposed to the light for brief periods [7]. It has also been used effectively to reduce damage from experimental pathology in a wide range of independent studies (see Table 1 in ref 7).

**Table 1:** The number of eyes used in separate experiments.

Experiment	670 nm (n)	Control (n)
RPE Flat mounts(1 eye)	5	5
Immunohistochemistry on sections (1 eye)	5	5
Western blots (1 eye)	4	5
qPCR (both eyes)	5	5

A total of twenty nine CFH^−/−^ mice were used for various applications.

Here we ask two questions. First, is 670 nm light a potential therapeutic route in an aged mouse model of AMD? Half of AMD cases are associated with polymorphisms of the complement system including complement factor H (CFH) [19]–[21]. There is a mouse model of CFH^−/−^ that has a distinct retinal phenotype with outer retinal disorganisation, elevated inflammation and reduced retinal function [22]–[24] that we employ here. Second, in previous studies using 670 nm light, animals have been held individually directly in front of the light source [7], [25]. Here we also ask whether 670 nm is therapeutic when given for short periods indirectly as part of the environmental lighting when animals are caged in groups and their retinae are not forcibly exposed to it individually. With both of these questions our analysis is focussed primarily on the outer retina, which is the location for disease initiation and progression in AMD, both in humans and in the mouse model [26]–[28].

## Materials and Methods

### Animals-ethics Statement

Twenty nine 16 month old CFH^−/−^ mice were used. Thirty nine eyes from these animals were used in different procedures (Table 1). Mice had been maintained from birth in a normal animal unit with a 12∶12 light cycle. None of the animals were exposed to direct lighting. All animals were used with University College London ethics committee approval and under a UK Home Office project licence (PPL 70/7036). All procedures were conducted in accordance to the United Kingdom Animal Scientific Procedures Act 1986.

### Therapeutic Light Treatment and Control

**Figure 1:**
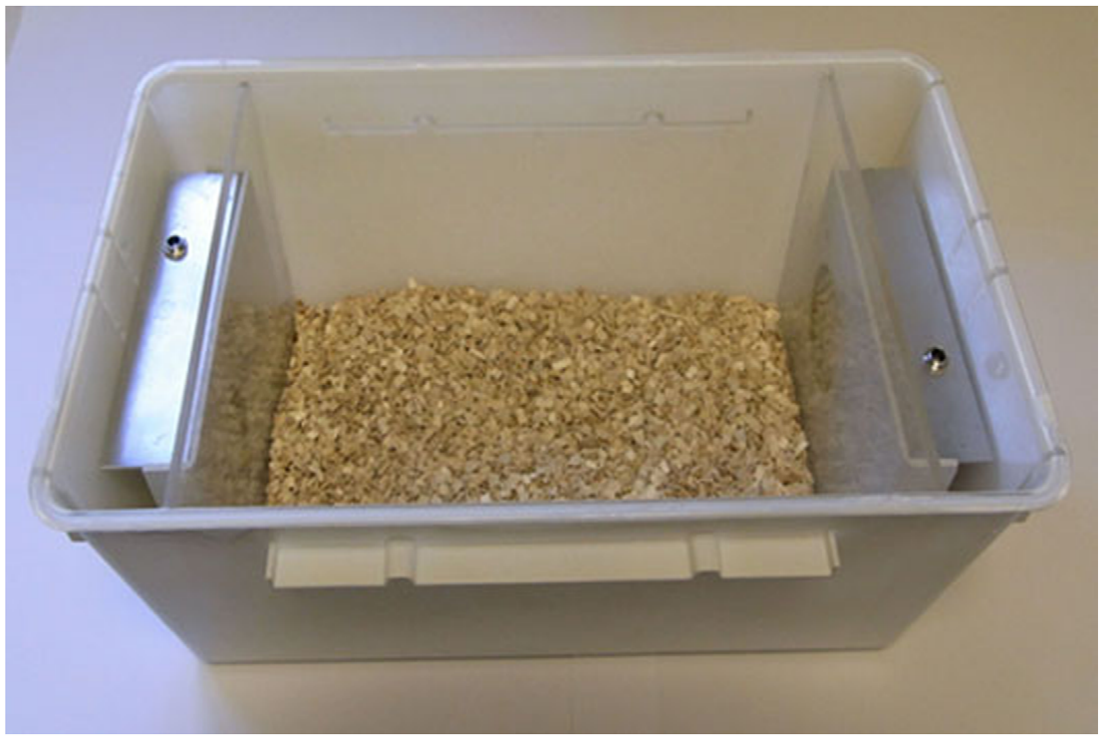
Cage for 670 nm exposure. LED lights were placed at both ends of the cage behind perspex screens. The internal space occupied a volume of 6,422 cubic centimeters (13 cm×26 cm×19 cm). A maximum of five animals were caged together. The longest distance the animals were from the light source was approximately 13 cm and the minimum was 0.4 cm, which is the thickness of the perspex screens.

The aged mice were divided randomly into two groups. These were caged separately for 14 days in adjacent compartments of a large animal cabinet. Individual cages had an internal space of 6,422 cubic centimeters, with a maximum of five animals per cage. The external walls of the cage were sprayed matt white to increase internal reflectance (Figure 1). All were exposed to low levels of room illumination on a 12∶12 L/D pattern of approximately 50 Lux.

**Figure 2:**
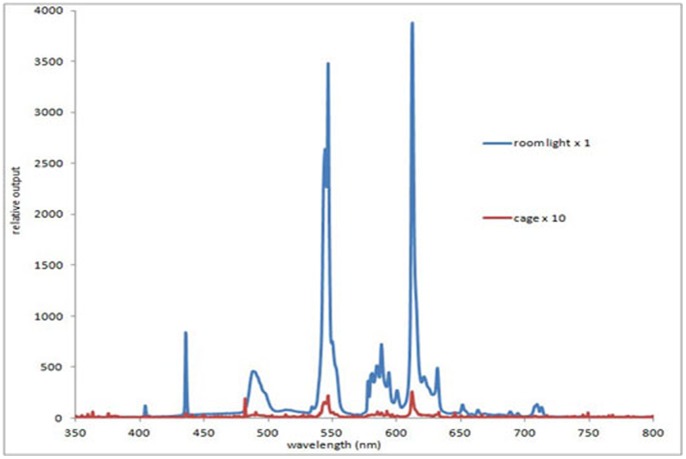
Spectral composition of room light in which mice were caged. The spectrum was measured directly under the room light which had an opaque cover (blue line) and again in the cage (red line), which did not receive any direct room illumination. The measurements for the cage are X 10 of those for the room light. As common with room lighting, it is primarily composed of a series of discrete peaks, however none of these are close to 670 nm.

The spectrum of the room illumination was measured at 0.5 m below the light source and within the cage with an Ocean Optics (USB2000+UV-VIS-ES, Dunedin, USA), showing minimal 670 nm content (Figure 2). Room illumination was indirect. The experimental group were exposed to 670 nm light via LED sources (C.H. Electronics, UK) behind clear perspex screens at either end of the cage (Figure 1). Spectral (Ocean Optics) and intensity measurements with a radiometer (International Lights, IL 1700 SED033IF/W, Massachusetts, USA) were made directly in front of the source and behind the perspex screens. There was no spectral shift as a consequence of the perspex screen and only vary minimal attenuation in intensity.

Energy levels for 670 nm and room lighting at different parts of the cage are given in Table 2. Each source produced 20 mW/cm^2^ and was turned on automatically for 6 minutes at approximately 6 am and 6 pm each day for 14 days. No attempt was made to modify the animal’s behaviour during exposures. The control group were housed separately, under identical conditions and could not see the 670 nm lights.

**Table 2:** Energy levels with 670 nm and room lighting.

	Energy levels (w/m^2^)	
Orientation	670 nm	room light
Cage floor (looking up)	0.85	2.25×10^−2^
Facing a light source	19	1.08×10^−2^
90° away from a light source	0.6	1.17×10^−2^

Following 14 days of light exposure all animals were killed by cervical dislocation and their eyes removed. Those for immunohistochemistry were fixed for 1 hour in 4% paraformaldehyde in phosphate buffered saline (PBS, pH 7.2) at room temperature, followed by X3 washes with PBS. Those for Quantitative real-time polymerase chain reaction (qPCR) and for Western blots were chilled on ice. Immunostaining was undertaken for a range of markers including COX, ionized calcium-binding adaptor molecule 1 (IBA-1), complement component C3, vimetin, glial fibrillary acidic protein (GFAP) and amyloid beta (Aβ). qPCR was used to confirm key COX and C3 labelling. Western blot analysis was undertaken additionally to confirm COX labelling as this is fundamental to the mode of action of 670 nm light.

### Immunostaining- Outer Retinal Macrophages

To examine the morphology of outer retinal macrophages flat mounts were stained with IBA-1. Eyes were dissected and flat mounts of the retinal pigmented epithelial (RPE) surface were produced following method used by Hoh Kam et al [2]. These were washed with PBS X1 for 5 minutes and blocked with 5% Normal Donkey Serum (NDS) in 3% Triton X-100 PBS for 2 hours on shaker. Followed by X1 wash with PBS and a primary antibody solution prepared by diluting 1% NDS in 3% Triton X-100 and incubated with IBA-1 (rabbit polyclonal, 1∶1000, A. Menarini Diagnostics, Wokingham, UK) overnight to mark macrophages. Next day the tissue was washed X3 with PBS and a secondary antibody solution prepared by diluting 2% NDS in 0.3% Triton X-100, incubated with donkey-anti rabbit 488 (1∶2000, Invitrogen, Paisley, UK) for 2 hours. Tissues were washed X3 with PBS then incubated with 4′,6-diamidino-2-phenylindole (DAPI, 1∶5000, Sigma Aldrich, Dorset, UK) for 1 minute in the dark to provide a counter stain. Lastly, tissues were washed several times with PBS and Tris buffered saline (TBS). The RPE flat mounts were mounted with Vectrashield (Vector laboratories, Peterborough, UK), cover slipped and sealed with nail varnish.

### Immunostaining- Sections

Fixed eyes were cryo-protected in 30% sucrose in PBS and embedded in optimal cutting temperature compound (OCT, Agar scientific, Stanstead, UK). Eyes were sectioned at 10 µm and thaw-mounted onto charged slides overnight. Immunohistochemistry was performed according to Hoh Kam et al [2]. Sections were initially washed for 5 minutes with PBS and then blocked with 5% NDS in 0.3% Triton X-100 solution for 1 hour. These were washed briefly and then a primary antibody solution was prepared by diluting 1% NDS in 0.3% Triton X-100 and incubated overnight. The following day, this was washed 1X with PBS, and a secondary antibody solution was prepared as described above and incubated for 1 hour. After incubation, this was washed several times and then DAPI was applied for 1 minute as a counter stain. Sections were then washed with PBS and TBS, mounted, cover slipped and sealed as described above. The following primary antibodies were used: COX subunit VIb (rabbit monoclonal 1∶200, Abcam, Cambridge, UK), C3 (rabbit polyclonal 1∶20, Abcam, Cambridge, UK), vimentin (rabbit monoclonal 1∶100, Abcam, Cambridge, UK), GFAP (mouse monoclonal 1∶1000, Abcam, Cambridge, UK) and Aβ (mouse monoclonal 1∶500, Covance, UK). This was followed by incubation with the appropriate Alexa fluor secondary antibody, donkey-anti rabbit 568 (1∶2000, Invitrogen, Paisley, UK) and donkey anti-mouse 568 (1∶2000, Invitrogen, Paisley, UK). Negative controls were done for all the above where the primary antibody was omitted.

### Quantitative Real-time Polymerase Chain Reaction

RNA was extracted using TRIzol reagent (Sigma Aldrich, Dorset, UK) from whole eye cups after the removal of extra-ocular tissue, cornea, lens and ciliary body/iris. The RNA was reverse transcribed using a Qiagen QuantiTect Kit and qPCR was performed using Power SYBR PCR master mix (Applied Biosystems, Paisley, UK) with the primer pairs; COX6B1 (Fwd: ACAATCTTTAGGAGTCAGGATGG, Rev: TTCTTAGTCTGGTTCTGGTTGG) and C3 (Fwd: GCGTAGTGATTGAGGATGGTG, Rev: ACAGTGACGGAGACATACAGG). Values were normalised using beta actin mRNA levels and analysed with DART-PCR software [29]. Each group consisted of 5 mice.

### Western Blot

Anterior chambers were dissected out leaving the retina and RPE-choroidal tissues, which were immediately frozen in liquid nitrogen and stored at −80°C. The tissues were homogenized in lysis buffer containing 4% sodium dodecyl sulfate, 17.5% glycerol, 0.25 M Tris and 100 mM DTT before extracts were electrophoretically separated on sodium dodecyl sulphate polyacrylamide gels. Subsequently, immunoblotted for COX (Abcam, Cambridge, UK) and tubulin (clone DM1A, Sigma Aldrich, Dorset, UK) as a housekeeping control protein. Changes of proteins were determined by densitometric scanning of immunoblots. Values were normalised to tubulin as loading control and averaged over at least 3 independent experiments [2], [30].

### Analysis

#### Macrophage number, morphology and distribution

Fluorescence images of the complete RPE surface were captured using a Nikon DMX1200 digital camera (Tokyo, Japan) at a magnification of X400 and saved in JPEG format to count macrophages. The numbers of IBA-1 positive cells per eye were determined using the count tool on Adobe Photoshop CS6.

To analyze macrophage morphology, images were captured at X1000 of cells that were clearly well labelled but still representative of the population. More labelled macrophages were commonly found in central regions approximately 1 mm from the optic nerve head than in other areas. The diameters of the whole mounts were approximately 5 mm with approximately 50 cells in experimental animals and about 90 cells in controls. Within each retinae 9–12 individual cells were selected for analysis on the basis of morphological clarity and a separation of more than 75 µm from any other cells analyzed. Within such constraints candidate cells were identified by scanning across the RPE surface.

For every image the total number of primary dendritic processes on each macrophage was counted by drawing a 30 µm diameter circle around the soma and then counting the processes crossing the circle, as undertaken by Lee et al [30]. The same images were used to measure primary process length from the centre of the nucleus to the tip of each dendrite. Dendritic field size was also determined using the same image. For this, the longest axis of the dendritic field was measured and that of the field orthogonal to this and the mean length calculated. Lasso tool in Adobe Photoshop CS6 was used to measure the cell body area on the same cells [30]. The distance between adjacent macrophages was calculated from nucleus of each cell to its nearest neighbour [2]. For this the images were taken at a lower magnification of X400, as was undertaken to count macrophage numbers.

#### Quantifying immunostaining intensity

The methods used to quantify the intensity of the immunostaining were similar to those employed previously [2], [7], [30]. Sections used for analysis had relatively uniform staining patterns. Two regions in the central retina were selected for analysis on either side of the optic nerve head that were approximately 200 µm away from the optic nerve and 150 µm wide and captured at a magnification of X400 in JPEG format. Pixel intensity was calculated in Adobe Photoshop CS6, by using the lasso tool to draw a line. No less than 10 measurements per eye were taken. The regions of interest for immune staining varied depending on the marker as they accumulate at different locations. For COX, this was at the level of the photoreceptor inner segments. For C3 it was Bruch’s membrane and photoreceptor outer segments, while for vimentin and GFAP measurements were made across the full depth of the neural retina. Aβ was measured along Bruch’s membrane/RPE interface. For all immunostaining sections from experimental and control groups were stained on the same day and imaged with standard microscope settings. All data were analysed with GraphPad Prism 5 and statistical analysis was undertaken using Mann-Whitney U non parametric tests.

## Results

### 670 nm Treatment Significantly Elevates COX Expression

**Figure 3:**
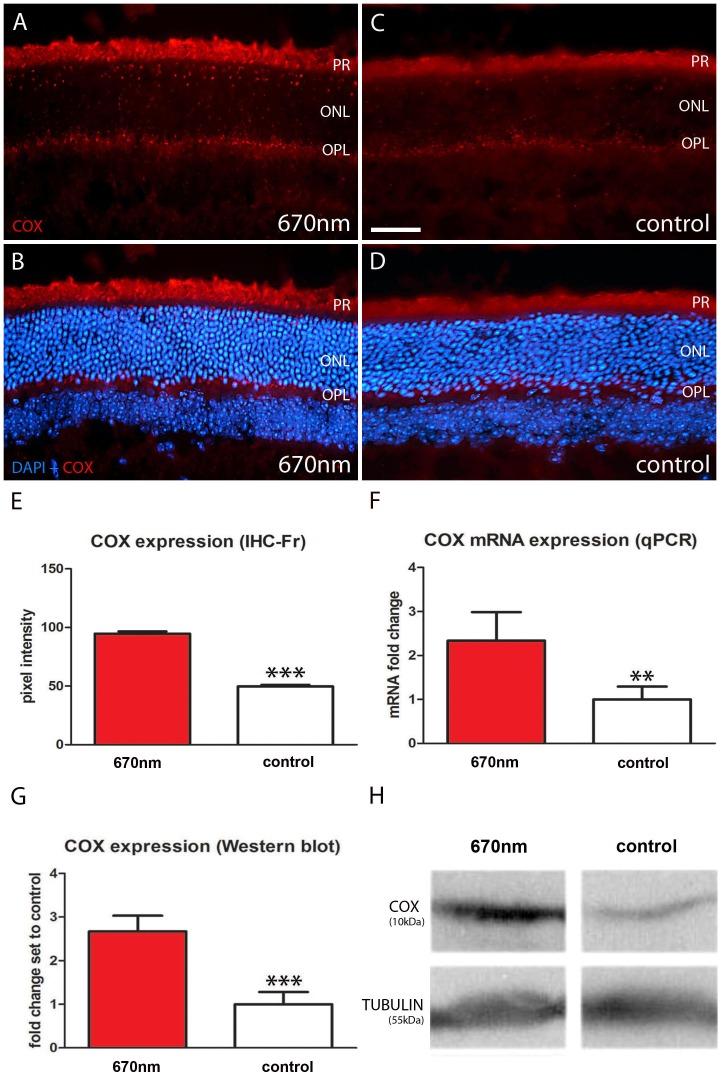
Mitochondrial cytochrome c oxidase expression is enhanced in 670 nm treated aged mice. A–D. Retinal sections were immunostained with COX (red). This enzyme is involved in mitochondrial respiratory chain and is activated by 670 nm light. In the experimental groups COX expression is significantly up regulated (A–B) compared to controls (C–E, p = 0.0001). B, D. Showed label with DAPI as a counter stain. COX staining was largely confined to photoreceptor inner segments and outer plexiform layer. F–G. This result was confirmed with significant increases in COX expression in qPCR (p = 0.0030) and Western blots (p = 0.0004). H. Differences for tubulin between groups were non-significant. Abbreviations, cytochrome c oxidase (COX), photoreceptor (PR), outer nuclear layer (ONL) and outer plexiform layer (OPL). Scale bar A–D = 40 µm.

COX is an enzyme in the mitochondrial respiratory chain which also functions as a photoacceptor molecule activated by long wavelengths [18], [25], [31]–[32]. COX immunostaining was present in both groups and was largely confined to mitochondrial rich regions, including photoreceptor inner segments and the outer plexiform layer (Figure 3A–D). There were significant differences between the two groups, in the experimental group COX expression was up regulated by approximately 50% (Figure 3E). To confirm the up regulation of COX following 670 nm treatment, quantitative Western blot and qPCR analysis were undertaken. These two independent methods confirmed similar significant increases with COX expression following light exposure (Figure 3F–H).

### 670 nm Treatment Significantly Changes Macrophage Morphology

**Figure 4:**
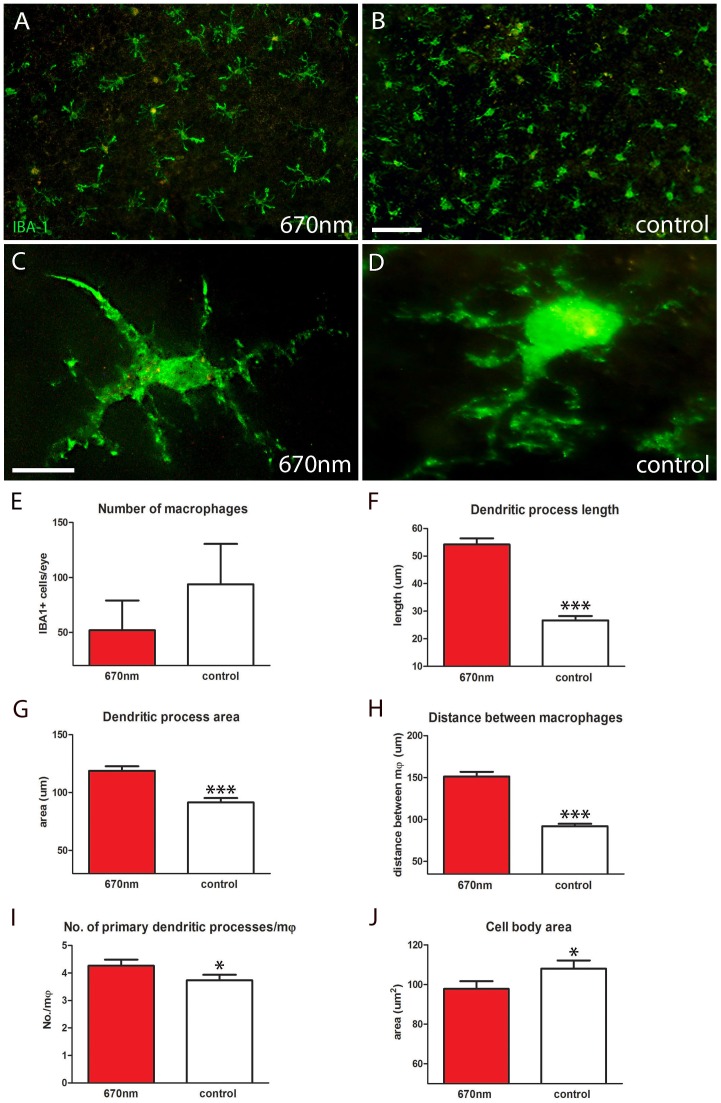
IBA-1 staining showed significantly different macrophage morphology between 670 nm and control groups. A–D. RPE flat mounts labelled with IBA-1 (green) to identify macrophages. After 14 days of treatment with 670 nm light these cells had significantly altered morphology over a range of metrics. E. Number of IBA-1^+^ cells per eye was measured; there was a reduction in the number of macrophages following treatment, but this was not statistically significant. F,G. Dendritic process length and area were significantly increased by 670 nm light (27%, 28% respectively) following treatment (p = 0.0001 for each). H. The distance between macrophages was measured from nucleus of one cell to its nearest neighbour, which also showed a significant increase (p = 0.0001). I. Not only were the 670 nm treated cells larger they also had more primary processes (p = 0.05). J. Even though these cells had a greater dendritic field and territory they had smaller cell bodies in comparison to controls (p = 0.05). Abbreviations, retinal pigmented epithelial (RPE), ionized calcium-binding adaptor molecule 1 (IBA-1), macrophages (mφ). Scale bars A, B = 40 µm, C,D = 20 µm.

IBA-1 is commonly used as a marker of macrophages. IBA-1 positive cells were widely distributed in a relatively uniform pattern over the RPE surface in both experimental and control groups, with somewhat more present in central than peripheral regions (Figure 4A–B). The morphology of the macrophages in the two groups appeared to be markedly different (Figure 4C–D) and although there were less stained cells in the light treated mice, this was not statistically significant (Figure 4E). However, the morphological differences between cells in the two groups were consistently significantly different over a range of metrics.

The primary processes in mice exposed to 670 nm light appeared to have wider bases and were significantly longer than those found in the control. In control mice mean primary process length was approximately 27 µm, while in experimental mice it almost doubled to approximately 54 µm (Figure 4F). Estimates for the area covered by the cells dendritic field between the two populations were consistent with this, as the longer processes in the 670 nm exposed mice covered a significantly larger area of the RPE surface (Figure 4G).

In the treated mice there was a significant increase in the distance between the cells from approximately 91 µm to approximately 151 µm (Figure 4H). Not only were the processes in treated mice longer, extending over a wider territory, but there were significantly more primary processes on them (Figure 4I). The relative expansion in the processes in 670 nm treated eyes was associated with a significant decline in the relative size of their cell bodies (Figure 4J). Hence, the dendritic architecture and size of individual macrophages in treated mice changed significantly in response to 670 nm light.

### 670 nm Treatment Reduces Outer Retinal Inflammation

**Figure 5:**
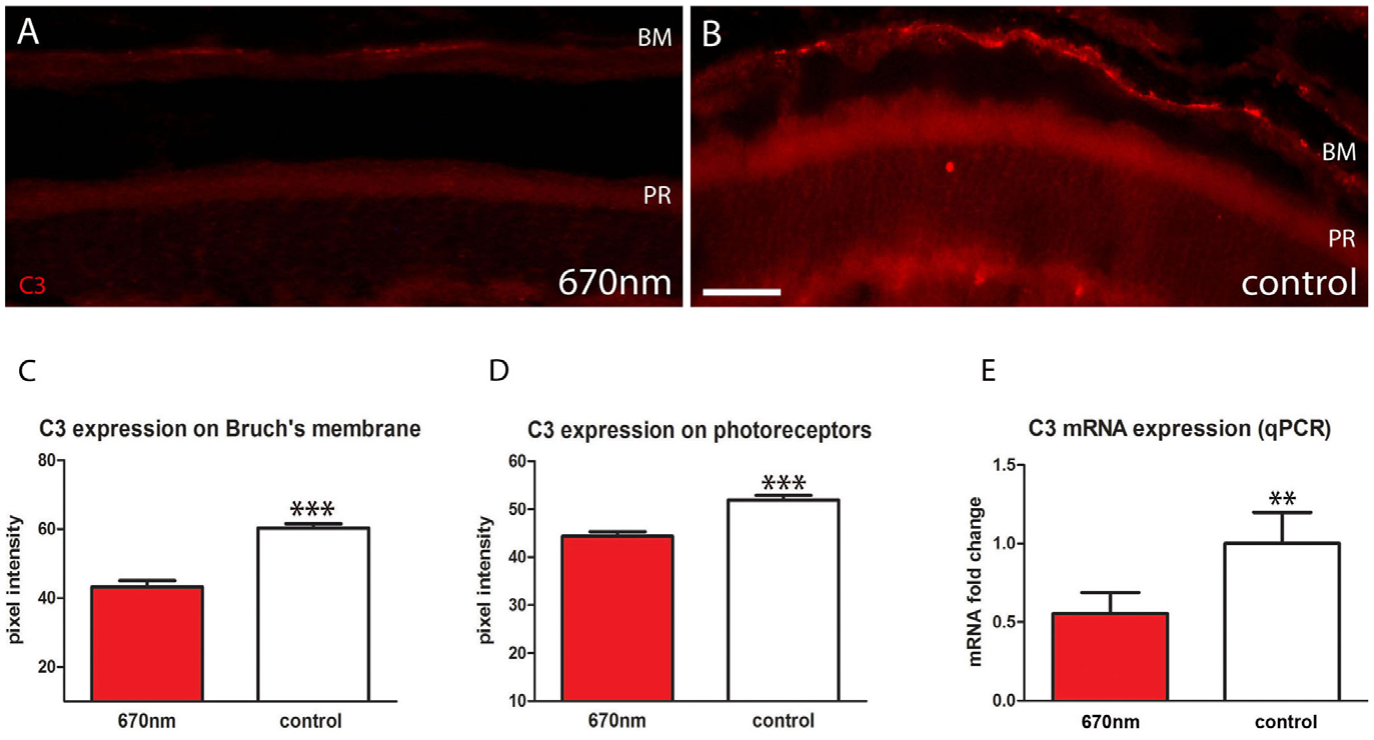
Outer retinal inflammation is significantly reduced following 670 nm treatment. A.B. Retinal sections stained with C3 (red). This accumulates on Bruch’s membrane and outer segments. C.D Following 670 nm treatment C3 was significantly reduced on Bruch’s membrane and photoreceptor outer segments (p = 0.0001 for each). E. These data were confirmed with qPCR analysis, which showed again a statistically significant reduction in C3 expression following treatment (p = 0.0031). Abbreviations, Bruch’s membrane (BM), photoreceptor (PR), complement component (C3). Scale bars = 40 µm.

The inflammatory marker C3 normally accumulates with age on Bruch’s membrane and on photoreceptor outer segments. C3 immunostaining was significantly lower in 670 nm treated mice at both locations than in controls (Figure 5A–D). On Bruch’s membrane it was almost halved, and on outer segments the reduction was approximately 20%. To confirm this finding C3 expression was also measured with qPCR, and again there was a significant reduction following 670 nm treatment with expression level halving (Figure 5E).

### 670 nm Treatment Reduces Retinal Stress

Retinal sections from the two groups were also stained for vimentin and GFAP. These are cytoskeletal intermediate filaments expressed in retinal Muller cells and are up regulated following retinal stress and ageing. Muller cell processes span the entire retina.

**Figure 6:**
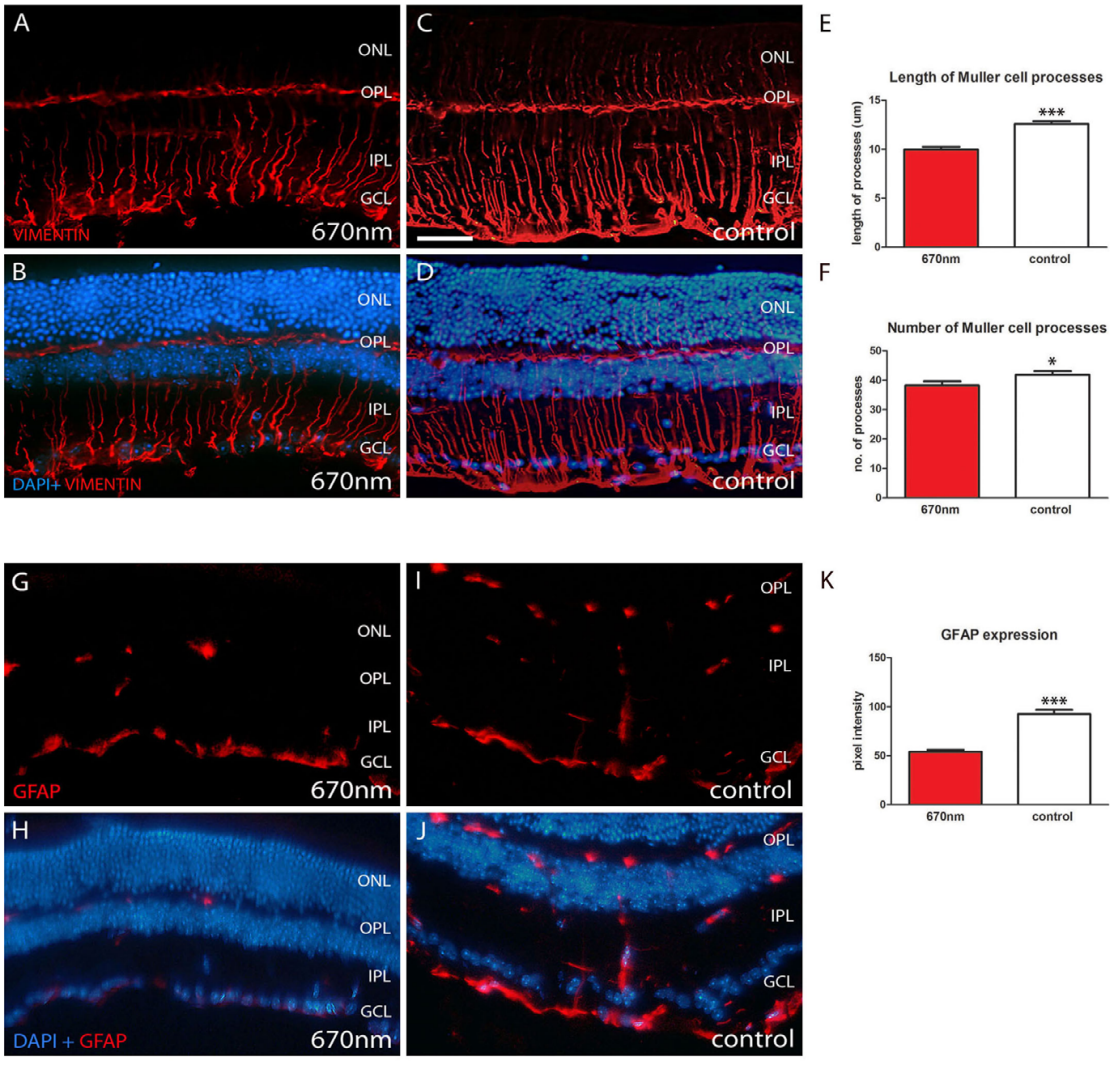
Retinal stress is significantly reduced following 670 nm treatment. Vimentin and GFAP are Muller cell markers, which are up regulated in ageing and when the retina is damaged or stressed. A–D. 670 nm treated groups showed significantly reduced vimentin labelling both in terms of length of Muller cells processes (E, p = 0.0001) and their number (F, p = 0.0329). G–J. Shows similar differences for GFAP with a statistically significant reduction following 670 nm treatment (K, p = 0.0001). In both A–D and G–J upper panels are immunostaining alone (red) while in lower panels DAPI is shown as a counter stain (blue). Abbreviations, outer nuclear layer (ONL), outer plexiform layer (OPL), inner plexiform layer (IPL), ganglion cell layer (GCL) and glial fibrillary acidic protein (GFAP). Scale bar A–D = 40 µm.

Staining was present for both vimentin and GFAP in both groups (Figure 6A–D, G–J). However expression of both was down regulated following 670 nm exposure. Vimentin labelling was much more extensive than GFAP in both experimental and control groups. With vimentin the number of Muller cell processes and their length were significantly reduced by 670 nm light (Figure 6E–F). In the untreated group (Figure 6C–D) labelling extended into the outer nuclear layer and was denser at the vitreal surface than in the treated mice. GFAP labelling was also significantly reduced following 670 nm light (Figure 6K), with more label present in the outer retina of controls (Figure 6I–J).

### 670 nm Treatment does not Alter Amyloid Beta Expression

**Figure 7:**
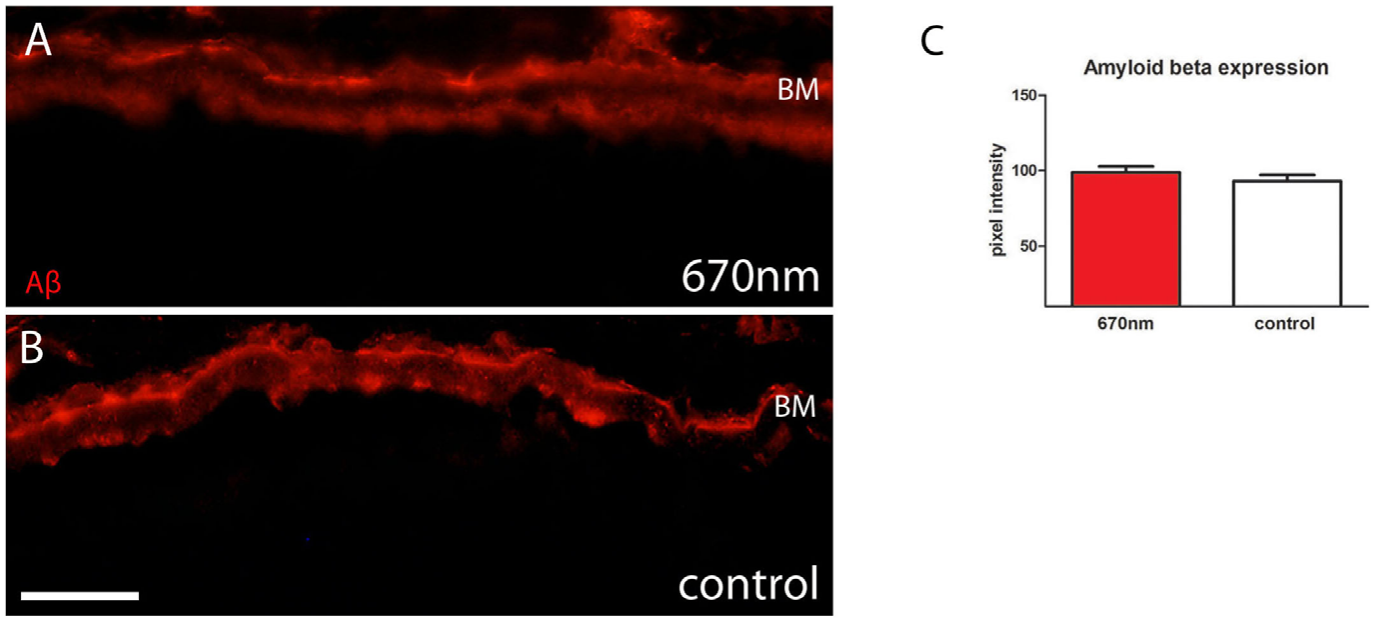
Amyloid beta expression is unaffected by 670 nm treatment. A.B. Aβ (red) expression was measured at the Bruch’s membrane/RPE interface and this was unchanged between experimental and control groups as shown in C. Abbreviation, amyloid beta (Aβ), Bruch’s membrane (BM), retinal pigmented epithelium (RPE). Scale bar A–B = 40 µm.

With age, the outer retina accumulates Aβ mainly on Bruch’s membrane and on photoreceptor outer segments [2]. This age related deposit is pro-inflammatory. Aβ was measured on Bruch’s membrane in both experimental and control group (Figure 7A–B). Measurements of staining intensity made at this interface showed no difference in deposition between the two groups (Figure 7C).

## Discussion

This study clearly shows that brief passive exposure to 670 nm light is effective in reducing inflammation in aged CFH^−/−^ mice in which retinal pathology has become established [23]. The retina showed marked changes after 670 nm treatment, with a significant increase in COX expression and significant reductions in C3, vimetin and GFAP expression. Further, there were significant changes in the dendritic morphology and cell body size of sub-retinal macrophages, although there number did not decline significantly. These changes occur independent of Aβ load, which did not differ between the two groups.

These results are similar to those that we obtained using normal aged C57BL/6 mice where retinal inflammation had become established [7]. However, in previous studies animals were individually held in front of the light in an attempt to regulate exposure [7]. This study shows that 670 nm light impacts therapeutically irrespective of the method of delivery and can be delivered by supplementing this wavelength in normal light, where it is largely absent.

While it is clear that a range of inflammatory/stress markers are reduced by 670 nm light similar to that shown by Kokkinopoulos et al [7], we did not find a statistically significant decline in macrophage numbers as found in this earlier study. However, it was obvious with other metrics that 670 nm light had a profound impact on these cells by simple observation. Another factor that significantly reduces inflammation and impacts on macrophage morphology is vitamin D. Here the number of cells is significantly reduced along with age related outer retinal inflammation. Further, the morphology of these cells also changes, but here they develop larger cell bodies and have fewer processes [30]. It is likely that reducing macrophages will be beneficial in aged tissue, but changes in their morphology in response to different therapeutic routes may be difficult to compare directly.

These results sit firmly within an expanding data set from different laboratories, consistently revealing that 670 nm exposure has a significant impact on both pathology and ageing within diverse tissues [17], [18], [25], [33]–[39]. Given this diversity, the mechanism of action must act upon a fundamental aspect of cell function. With ageing and many forms of pathology, mitochondrial DNA is damaged and this probably reduces ATP output, which is critical for normal metabolic function. Changes in mitochondrial function are key to theories of ageing as the driving force for ageing probably relates to mitochondrial DNA damage reducing ATP and increasing ROS output, which is inflammatory and induces tissue degradation and cell loss [40]. The impact of such damage is marked in regions were metabolic rate is great, as in the outer retina [4]–[6]. Age related changes here not only include increased inflammation and deposition, but also the loss of approximately 25–30% of central photoreceptors in both man and mouse [13], [41].

The exact mechanism of 670 nm has not been proven. However it has been proposed that this wavelength is selectively absorbed by COX in mitochondria [42]. COX is the rate limiting enzyme in mitochondrial respiration, which we have shown is significantly up regulated following 670 nm exposure. Its activation enhances the efficiency of oxidative phosphorylation, which may in turn lead to an increase in ATP production and a reduction in ROS [17], [18], [37], [43], [44]. This in turn may reduce oxidative stress in the outer retina, which due to very high metabolic demand is particularly susceptible to this type of damage [4]–[6]. Whether this impacts on transcription factors and gene expression is unknown and there remains considerable speculation regarding these issues [31], [45]. However our current in vivo studies have confirmed that retinal ATP production declines with age and that 670 nm exposure corrects this.

Dosages of 670 nm vary between studies, although they tend to be relatively brief, but efficacy is common, indicating that the actual exposure levels required may be lower than had been expected. This is consistent with our data, as exposure levels must have varied between animals because they were often asleep through exposures, while at other times they were active but not necessarily oriented towards the source of the 670 nm light. In spite of this, because of the relatively long wavelength of 670 nm, it will penetrate deeply into the tissue. Consistent with this was the observation that the light from our delivery devices could be seen through the hand in a dimly lit room. Further, in a study this wavelength has been shown to reduce the pathology from partial section of the rat optic nerve when illumination was over the top of the head and only a very small amount of the light actually penetrated skull tissues [34]. Hence, 670 nm appears to be effective relatively independent of dosage or at least over a very wide range. However, we do not know a number of key variables, including how long following an exposure the impact of 670 nm remains effective, and also whether excess exposure ultimately has a negative impact or is ineffective. Resolution of these questions remains critically important in steps towards human therapeutic treatment.

In both normal ageing and in AMD outer retinal deposits and inflammation develop, partly due to the high metabolic demand of photoreceptors and the accumulation of relatively large quantities of extra-cellular debris, including Aβ, the heavier elements of which are pro-inflammatory [2], [26], [46]–[48]. However, here we demonstrate that it is possible to disassociate some of these factors by reducing markers of inflammation/stress independent of Aβ load. While there is little doubt that Aβ is toxic [49], [50], this is mainly true of the larger molecular forms, as the smaller elements are actually critical for synaptic plasticity and normal central nervous system function [51], [52]. Eventually, it is likely that progressive Aβ deposition on Bruch’s membrane will significantly compromise the permeability of this interface between the outer retina and its blood supply, which will probably impact negatively on outer retinal function. However, it is unclear to what extent such changes drive neuronal degeneration compared with inflammation alone. A key strategy in fighting retinal ageing is focussed on the issue of clearing Aβ systemically, which in turn is hoped to impact on inflammation. However, physiological levels of Aβ are necessary and their removal is likely to be detrimental with a loss of function [51], [52]. An alternative route that would avoid such problems may reside in the results of the study presented here.

Therapeutic routes in AMD have already been initiated using 670 nm light. While the patient numbers have been very low, they have shown significant improvements in terms of visual function in some using devices that were held directly in front of the eye [53]. While patient numbers need to be expanded and followed over long time periods, such results linked with this study and our previous work are encouraging.
